# Integrated transcriptome and hormone analyses provide insights into silver thiosulfate-induced “maleness” responses in the floral sex differentiation of pumpkin (*Cucurbita moschata* D.)

**DOI:** 10.3389/fgene.2022.960027

**Published:** 2022-08-29

**Authors:** Qingfei Li, Peiwen Chen, Hao Tang, Fansen Zeng, Xinzheng Li

**Affiliations:** ^1^ College of Horticulture and Landscape, Henan Institute of Science and Technology, Xinxiang, China; ^2^ Henan Province Engineering Research Center of Horticultural Plant Resource Utilization and Germplasm Enhancement, Xinxiang, China

**Keywords:** silver thiosulfate, sex differentiation, transcriptome, hormone, pumpkin

## Abstract

The perfect mating of male and female flowers is the key to successful pollination. The regulation of ethylene with chemicals is a good option for inducing staminate or female flowers. Silver thiosulfate is often used to induce the formation of male flowers in subgynoecious and gynoecious crops, which is important to maintain their progenies. However, its effects on flower sex differentiation in pumpkin (*Cucurbita moschata* Duch.) and the underlying mechanism remain unclear. In this study, the application of silver thiosulfate to pumpkin seedlings significantly delayed the occurrence of the first female flower and increased the number of male flowers. We next investigated the underlying mechanism by employing transcriptome and endogenous hormone analyses of the treated plants. In total, 1,304 annotated differentially expressed genes (DEGs)were identified by comparing silver thiosulfate-treated and control plants. Among these genes, 835 were upregulated and 469 were downregulated. The DEGs were mainly enriched in the phenylpropanoid biosynthesis and metabolism pathways (phenylalanine ammonia-lyase, peroxidase) and plant hormone signal transduction pathways (auxin signaling, indole-3-acetic acid-amido synthetase, ethylene response factor). Silver thiosulfate significantly reduced the levels of 2-oxindole-3-acetic acid, para-topolin riboside, dihydrozeatin-O-glucoside riboside, and jasmonoyl-l-isoleucine but increased the levels of trans-zeatin-O-glucoside, cis-zeatin riboside, and salicylic acid 2-O-β-glucoside. The levels of auxin and jasmonic acid were decreased, whereas those of salicylic acid were increased. Different trends were observed for different types of cytokinins. We concluded that silver thiosulfate treatment not only affects the expression of auxin synthesis and signaling genes but also that of ethylene response factor genes and regulates the levels of auxin, salicylic acid, jasmonic acid, and cytokinins, which together might contribute to the maleness of pumpkin. This study provides useful information for understanding the mechanism underlying the effect of silver thiosulfate on floral sex differentiation in pumpkin, a widely cultivated vegetable crop worldwide, and gives a production guidance for the induction of maleness using STS for the reproduction of gynoecious lines of Cucurbitaceae crops.

## Introduction

Most Cucurbitaceae plants exhibit monoecy, possessing unisexual female and male flowers in a single plant. Pumpkin (*Cucurbita moschata* D.), a typical monoecious plant, is a widely cultivated vegetable crop worldwide (Ma et al., 2022). Pumpkin is popular among Chinese people, and China ranks first in total annual output of pumpkins throughout the world. Only when the female and male flowers open at the same time can pollination be completed perfectly, with subsequent ovary enlargement and fruit formation. However, there are often no suitable female flowers when the male flowers are open, or there are no male flowers to provide mature pollen when the female flowers are about to open. Especially in gynoecious and subgynoecious plants, which bear only female flowers or few male flowers, there are often no suitable male flowers to provide mature pollen. Thus, in many cases, sex modification with chemicals is necessary.

Ethylene is a key regulator of floral sex differentiation and has been used to induce female flowers in many species of *Cucurbita* and *Cucumis* (Jin et al., 2011; Li et al., 2021). At present, many genes regulating sex determination, via direct or indirect means, have been found to be involved in ethylene functions. Ethylene mediates interactions among different sex-regulating genes, such as *1-aminocyclopropane-1-carboxylate synthase* (*ACS*), *1-aminocyclopropane-1-carboxylic acid oxidase* (*ACO*), a transcription factor (*WIP1*, domains containing the sequence Trp-Ile-Pro), and *ethylene responsive factor* (*ERF*) (Pan et al., 2018; Houben and Van de Poel, 2019; Li et al., 2022). In addition to the ethylene biosynthesis genes, ethylene perception and signaling genes play important regulatory roles in floral sex differentiation. Inhibiting the expression of *CsETR1*, encoding one of the ethylene receptors, induces DNA damage in primordial anthers and arrests stamen development in female flowers (Duan et al., 2008; Wang et al., 2010). Furthermore, mutants of *etr1a* and *etr2b* (two ethylene receptor) exhibit altered sex determination and the conversion of female flowers into bisexual or hermaphrodite flowers in *Cucurbita pepo* (García et al., 2019).

The application of anti-ethylene agents is a much easier and faster way of inducing male flowers; these include the ethylene perception inhibitors silver thiosulfate (STS) (Veen, 1983) and silver nitrate (AgNO_3_) (Kumar et al., 2009) and the ethylene biosynthesis inhibitor aminoethoxyvinylglycine (AVG). Thus, blocking ethylene responses is an efficient method to restrain some of the undesirable effects of ethylene. Several studies have reported the effect of anti-ethylene agents on crops. Floral sex differentiation can be regulated by the application of AgNO_3_ solution (200–500 mg/L), which reduces the number of female flowers in monoecious cucumber and increases the number of staminate flowers in gynoecious plants (Owens et al., 1980; Stankovic and Prodanovic, 2002). Especially, the production of more seeds per fruit was achieved when pollen from staminate flowers induced with high concentrations of AgNO_3_ was used to pollinate pistillate flowers of another gynoecious cultivar (Koyama, 2008). Moreover, AgNO_3_ treatment leads to androgenesis in gynoecious cucumber plants (Amirian et al., 2020), and its application enhances the growth of stamens in female flowers of *Coccinia grandis* (Ghadge et al., 2014). In dioecious plants of Cucurbitaceae, namely female plants of kakrol (*Momordica dioica* Roxb.) and pointed gourd (*Trichosanthes dioica* Roxb.), AgNO_3_ treatment results in the development of hermaphrodite flowers (Parimala et al., 2019), and pollen from induced hermaphrodite flowers results in the production of normal fruits and seeds (Hassan and Miyajima, 2019). In addition to floral sex differentiation, ethylene inhibitors also affect the node position of flowers. Treatment with AgNO_3_ and AVG decreases the nodes of male/bisexual flowers on the main stem of melon (Ye et al., 2020).

In addition to AgNO_3_, STS is another ethylene perception inhibitor. In the gynoecious plants of bitter gourd, STS induces the development of hermaphrodite flowers in the staminal tissue of female flowers, and these effects were found to be better than those of low concentrations of AgNO_3_ and GA_3_ (Mishra et al., 2015). Foliar sprays of STS induce the production of male organs in female hemp plants (Lubell and Brand, 2018). Comparative RNA-Seq analysis of untreated and STS-treated female *Cannabis sativa* revealed that many differentially expressed genes (DEGs) are transcription factors, and several others are involved in male organ development, phytohormone signaling, and male-biased phenotypes associated with masculinization (Adal et al., 2021).

Ethylene sometimes causes undesirable effects, such as the ageing and wilting of flowers, in ornamental plants. Thus, besides its effect in floral sex differentiation, blocking ethylene responses is a practical method to prolong the longevity of flowers in plants that are sensitive to ethylene (Serek et al., 2015). STS blocks the ethylene surge before the wilting of petals by blocking the “receptor site” for ethylene (Veen, 1983). In addition, STS can be used to increase the number and longevity of flowers in cassava, and the beneficial effects of STS are restricted to the shoot apex tissues (Hyde et al., 2020). Moreover, the application of STS, in combination with an extended photoperiod, effectively induces flowering with male fertile flowers in potato (Kumar et al., 2006). Furthermore, treatment with STS and nano-silver prolongs the longevity of several cut flowers. STS and nano-silver also alleviate the effects of ethylene and extend the vase life of cut carnation flowers (Liu et al., 2018).

Although STS is an effective ethylene perception inhibitor, its effects on flower sex differentiation in pumpkin and the underlying mechanism remain unclear. In this study, we assessed the effects of STS application on flower sex determination in pumpkin and explored the regulatory mechanism by assessing the transcriptome and determining the levels of hormones in the shoot apical meristems after STS treatment.

## Materials and methods

### Plant materials and sampling

An inbred line of pumpkin, “009-1” (obtained by more than 10 generations of selfing and stored in our laboratory), was used in this experiment. The seeds were germinated in a glass Petri dish, which was lined with a moist filter paper. After the seeds germinated, they were transferred to a mixed substrate, consisting of 66.7% (v/v) peat moss, 22.2% (v/v) vermiculite, and 11.1% (v/v) perlite, and grown under natural light with a daily average temperature of 21.5 ± 3.3°C (Xinxiang city, China, lat. 35° 16′ 58″N, long. 113° 55′ 44″E). The shoot apices of seedlings were sprayed with 600 mg/l of STS solution containing 0.1% Tween-20 (STS treatment) and 0.1% Tween-20 (control) twice every 2 days, when the third true leaves of the seedlings unfolded. The concentration of STS was chosen referring to previous studies (Stankovic and Prodanovic, 2002).

The shoot apical meristems were collected for RNA-Seq analysis and quantitative real-time PCR (qRT-PCR) validation; when they were exposed to STS for 4 h, no significant effect on gene expression was found within 4 h of STS treatment in a preliminary experiment. The content of hormones was determined 1 day after the second treatment with STS. The samples were collected and put into liquid nitrogen immediately for quick-freezing and then stored at −80°C for subsequent tests. RNA-Seq, qRT-PCR, and hormone analyses were performed with three biological replicates, and one biological replicate comprised a pooled sample of shoot apical meristems from three plants.

### Floral sex differentiation

The number of male and female flowers located at the top 25 nodes of each plant was counted. The node representing the occurrence of the first female flower was recorded. To avoid the duplicate or missed counting of flowers, the nodes were marked with a green line after every survey. At least six plants were used to assess the effects of STS on sex differentiation in pumpkins.

### RNA isolation and sequencing

RNA was extracted from frozen shoot apical meristems subjected to STS and control treatments using the MiniBEST Plant RNA Extraction Kit (Takara, Dalian, China). The purity of RNA was checked using the NanoPhotometer^®^ spectrophotometer (Implen, CA, United States), and its concentration was measured using a NanoDrop DU8000 (Thermo, CA, United States). The Agilent 2100 system (Agilent, CA, United States) was used to assess the RNA integrity, with a minimum RNA integrity number of 9.0. Good quality RNA from three biological replicates was used to build cDNA libraries by Biomarker technologies (Beijing, China). The high-quality libraries were sequenced with an Illumina HISeq 2500 platform, and paired-end reads were generated.

The low-quality and adaptor sequences were removed from the raw reads. Clean reads were then mapped to the *C. moschata* genome using TopHat2 (Kim et al., 2013; Sun et al., 2017), allowing for up to one mismatch. Differential expression analysis was conducted using the DESeq R package. The expression levels of genes were represented by the fragments per kilobase of transcript per million fragments value. *p*-values were adjusted using the Benjamini and Hochberg’s method (Benjamini and Hochberg, 1995) for controlling the false discovery rate. Genes with an adjusted *p*-value < 0.05 and a fold change ≥1.5 were considered as differentially expressed.

Gene functions were annotated using the NR, Nt, Pfam, KOG, Swiss-Prot, Gene Ontology (GO), and Kyoto Encyclopedia of Genes and Genomes (KEGG) databases.

### Functional enrichment analysis of DEGs

The GOSeq R package (Young et al., 2010), which can adjust for gene length bias in DEGs, was used to perform GO enrichment analysis. All genes in the reference genome and new genes were used as the background gene set in the GO over-representation analysis. The KEGG pathway enrichment analysis was implemented using KOBAS software (Mao et al., 2005). A *p*-value for DEGs ≤0.05 was considered significant enrichment in GO and KEGG pathway enrichment analyses.

### Quantitative real-time PCR validation

The reliability of expression levels obtained from RNA-Seq was confirmed using qRT-PCR. First-strand cDNA was synthesized using the PrimeScript^™^ RT Master Mix (Perfect Real Time) Reagent Kit (Takara, Dalian, and China). qRT-PCR was performed using Takara SYBR Premix Ex Taq™ and run on Bio-Rad IQ5 equipment (Foster City, CA, United States). The expression levels were calculated with the reference gene *ACTIN* using the 2^−ΔΔCt^ method (Livak and Schmittgen, 2001). The gene names and sequences of primers are detailed in Supplementary Table S1. The expression levels were determined using three technical and three biological replicates. Correlation analysis of the log_2_ (fold-change) values obtained in RNA-Seq and qRT-PCR was carried out to assess the reliability of transcriptome data.

### Hormone analysis

The shoot apical meristems (50 mg, fresh weight), which were frozen in liquid nitrogen, were ground to powder for use. The quantification of endogenous hormones was performed by Wuhan Metware Biotechnology Co., Ltd. (Wuhan, China) with an LC–MS/MS platform as described by Guo et al. (2021). The extracts were analyzed using a UPLC-ESI-MS/MS system. For MS/MS analysis, an AB 6500 + QTRAP^®^ LC-MS/MS System was used. Different concentrations of standard solutions (0.01, 0.05, 0.1, 0.5, 1, 5, 10, 50, 100, 200, and 500 ng/ml) were used to generate standard curves for each hormone.

### Statistical analysis

IBM SPSS Statistics 22 and Microsoft Excel 2010 software were used to analyze the data, determine the variances, for correlation analysis, and to prepare the diagrams. Multiple comparisons (*p* < 0.05) were conducted using Duncan’s new multiple range test. Values were expressed as means ± standard errors (SEs).

## Results

### Effects of STS on floral sex differentiation in pumpkin

**FIGURE 1:**
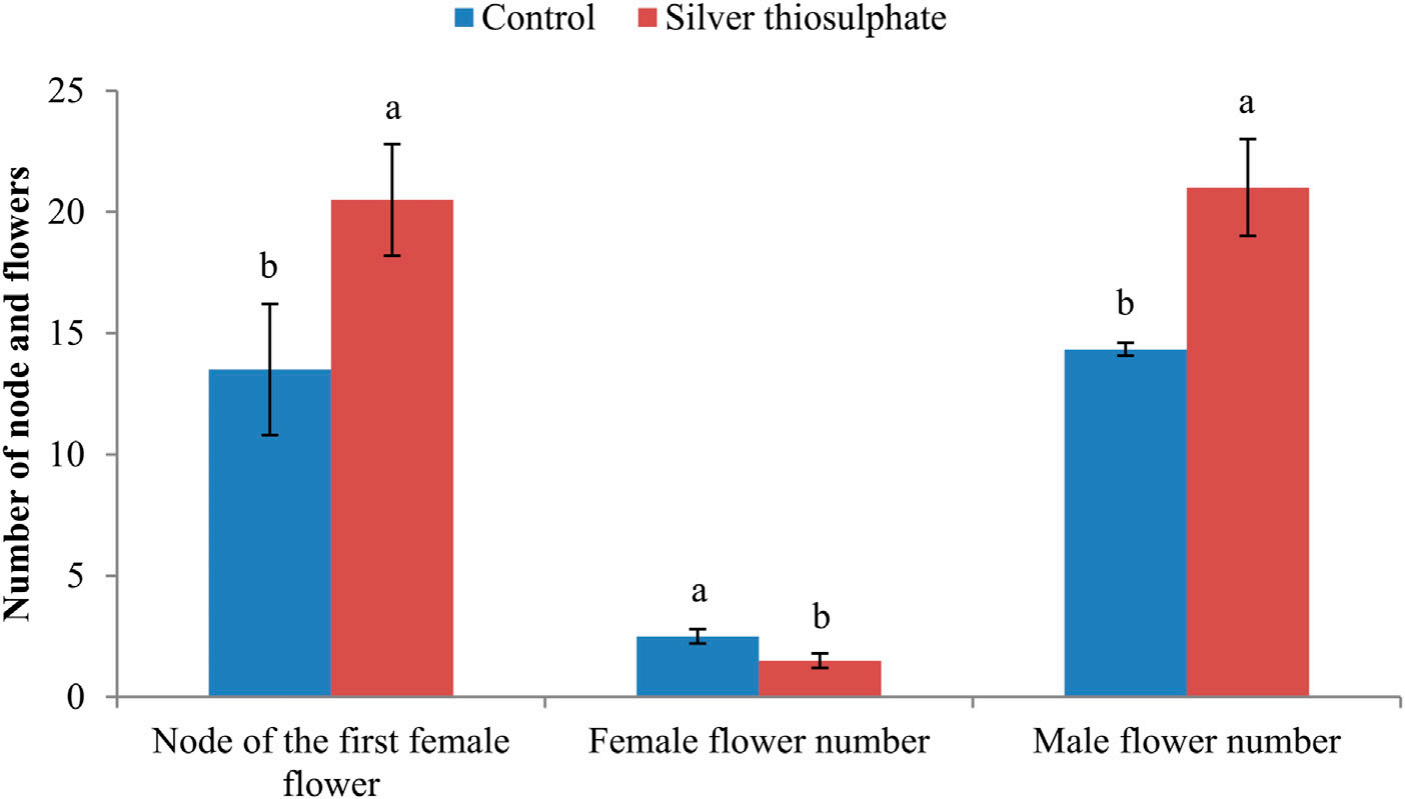
Effect of silver thiosulfate on the node of the first female flower and the number of female and male flowers. The lowercase letters represent a significant difference between control and silver thiosulfate-treated plants at the 5% level, as determined by Duncan’s new multiple-range test.

Floral sex differentiation in the top 25 nodes of pumpkin plants was investigated. STS treatment had obvious effects on the node of the first female flower and on the number of female and male flowers. STS treatment significantly delayed the appearance of the first female flower from node 13.50 ± 2.70 (in the control) to node 20.50 ± 2.30. The number of male flowers within the 25 nodes in STS-treated plants (21.00 ± 2.00) was significantly higher than that in the control plants (14.33 ± 0.27). STS treatment reduced the number of female flowers from 2.50 ± 0.30 to 1.50 ± 0.30 (Figure 1).

### RNA-Seq data analysis

**FIGURE 2:**
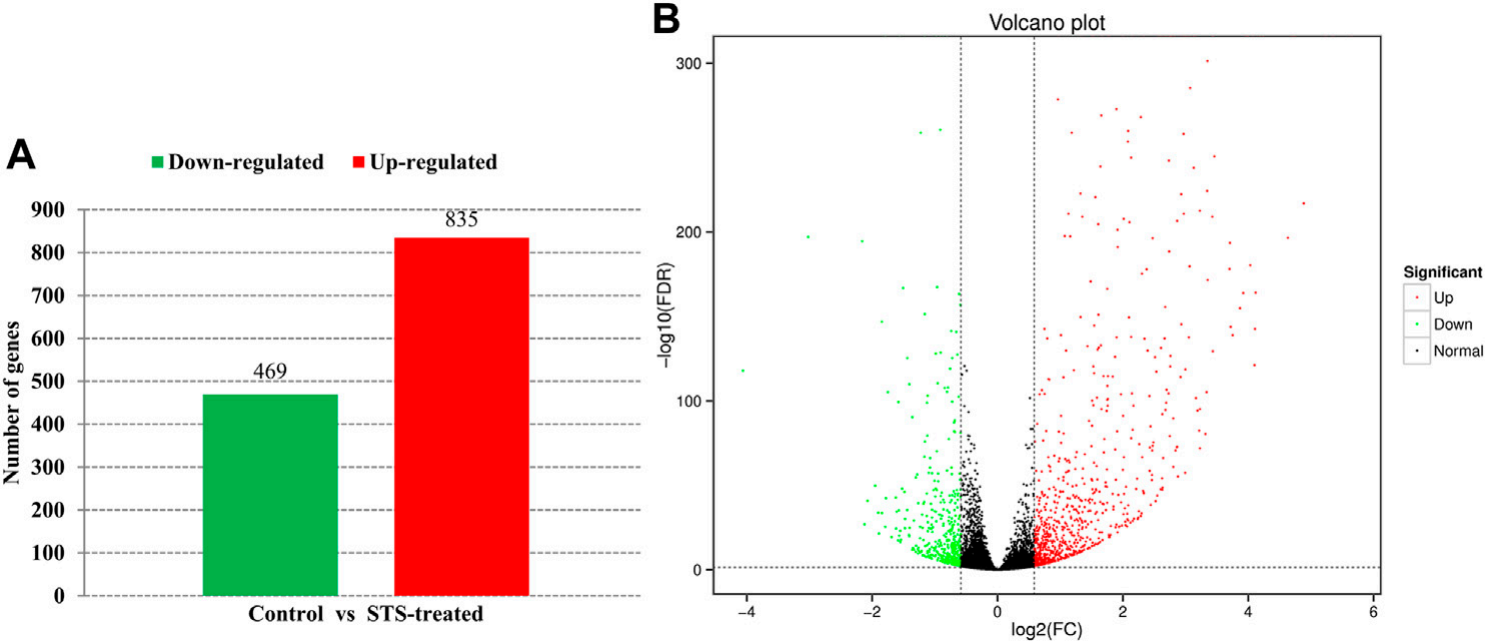
Differentially expressed genes (DEGs) between silver thiosulfate (STS)-treated plants and control plants. **(A)** Numbers of upregulated and downregulated genes. **(B)** Volcano map of the gene expression.

An average of 46,013,742 clean reads was obtained, and the percentage of clean reads that mapped to the *C. moschata* genome ranged from 94.95% to 95.56%. The Q30 quality score of each sample was not less than 93.03%. The RNA-Seq data of each sample are detailed in Supplementary Table S2. In total, 1,304 annotated DEGs, which included 469 downregulated and 835 upregulated genes, were identified by comparing the transcriptomes of STS-treated and control plants (Figure 2).

### Functional enrichment of DEGs induced by STS

**FIGURE 3:**
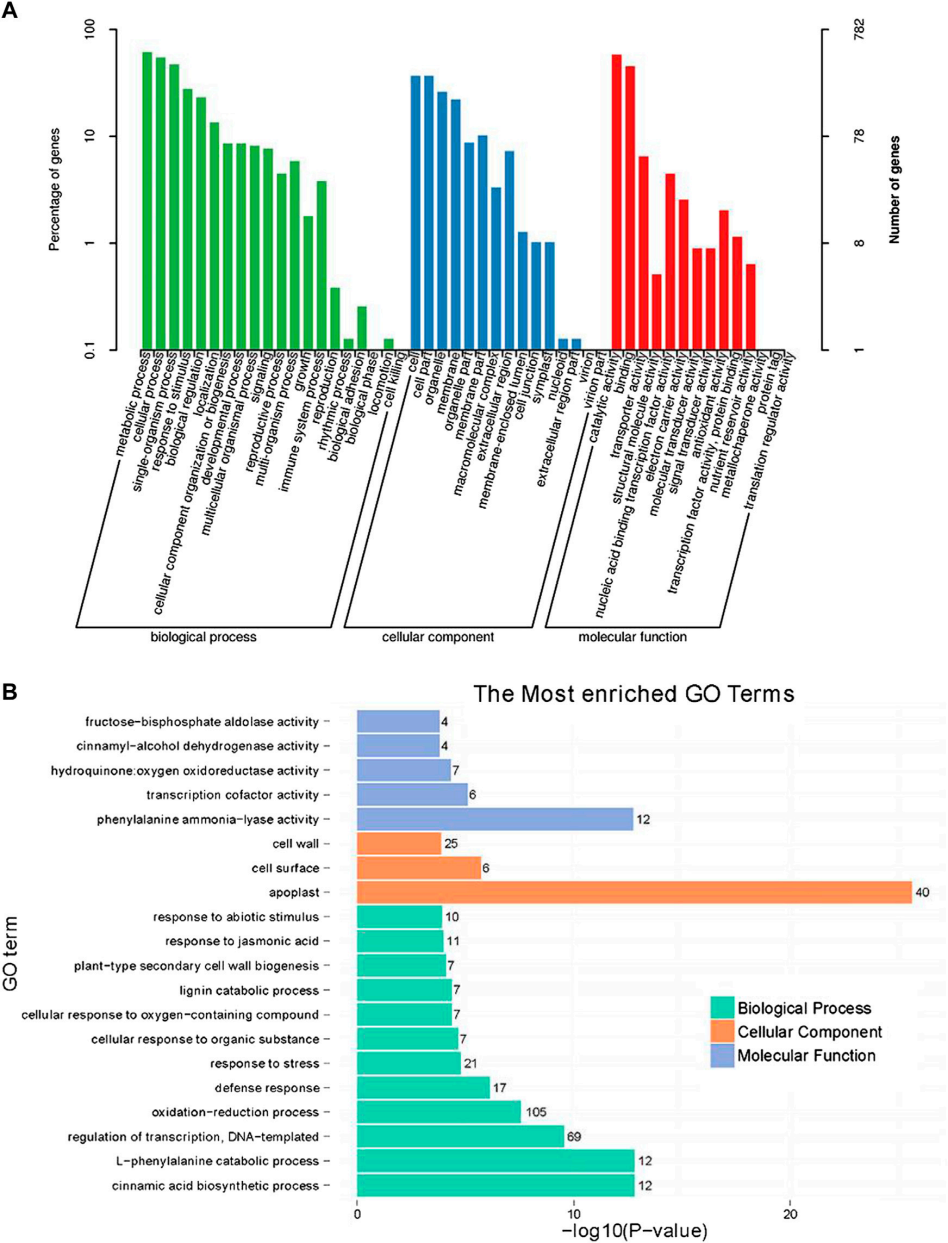
Gene ontology (GO) analysis of differentially expressed genes (DEGs) between shoot apical meristems of control and silver thiosulfate-treated plants. **(A)** GO functional categories. **(B)** Enrichment analysis of DEGs based on molecular function, cellular component, and biological process categories. The figures on the left of each term represent the number of DEGs.

GO functional classification indicated that the annotated genes were assigned to three major functional categories, including 49 GO terms at the annotation level 2, specifically biological processes (20 terms and 616 genes), cellular components (15 terms and 381 genes), and molecular functions (14 terms and 643 genes) (Figure 3A). In the molecular function category, the most significantly enriched GO term was phenylalanine ammonia-lyase activity (GO:0045548). In the cellular component category, the most significantly enriched GO term was apoplast (GO:0048046), which indicated that the proteins encoded by the DEGs might function in the apoplast. In the biological process category, the most significantly enriched GO term was the cinnamic acid biosynthetic process (GO:0009800), and it included the same DEGs as the l-phenylalanine catabolic process (GO:0006559) term (Figure 3B).

**FIGURE 4:**
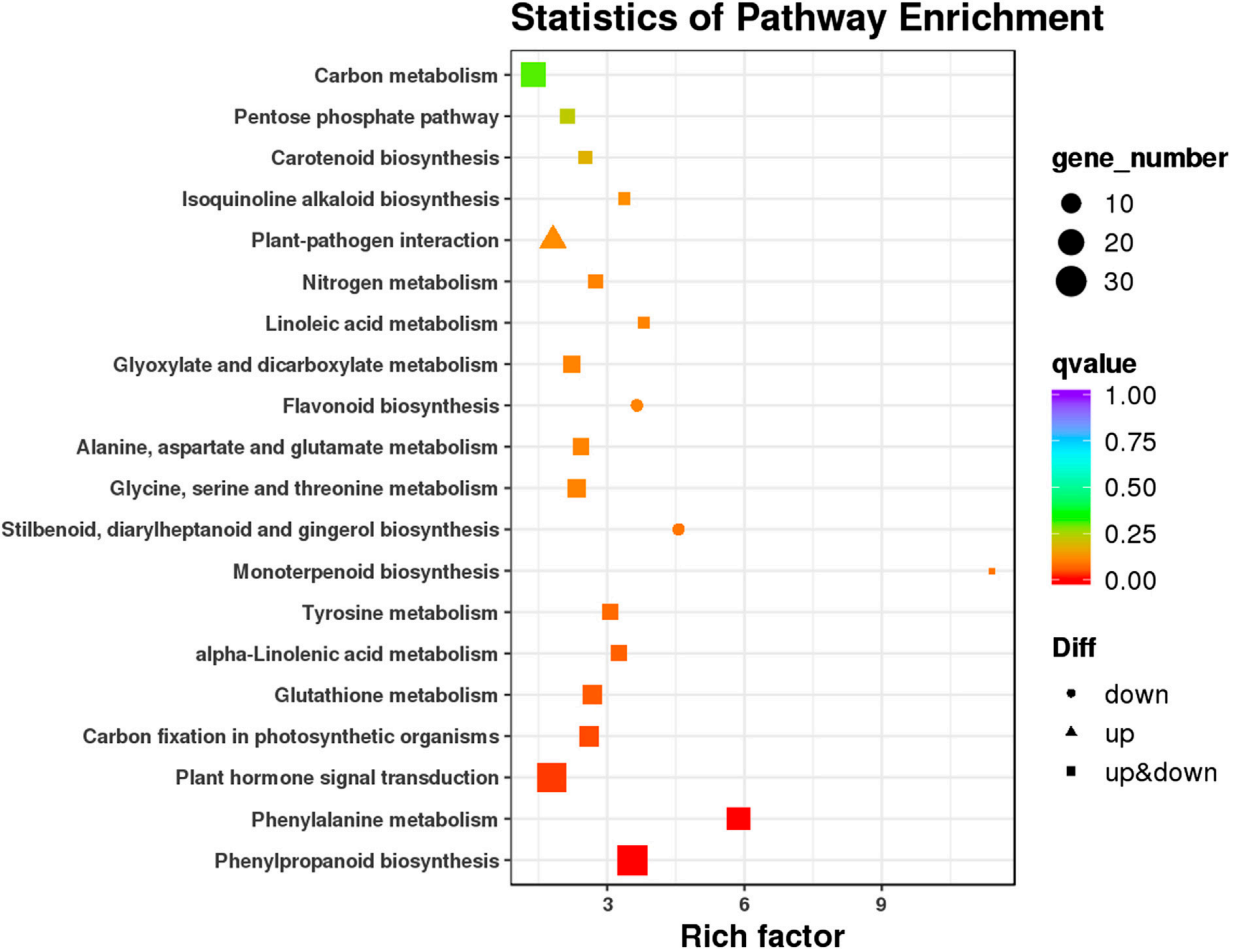
Statistics of Kyoto Encyclopedia of Genes and Genomes (KEGG) pathway enrichment analysis.

The results of KEGG pathway analysis showed that 239 DEGs could be assigned to 87 KEGG pathways. The significantly enriched KEGG pathways are as shown in Figure 4. Many DEGs were involved in phenylpropanoid biosynthesis (33 DEGs), phenylalanine metabolism (17 DEGs), and plant hormone signal transduction pathways (30 DEGs). All but three DEGs that were involved in the phenylpropanoid metabolism pathway belonged to the phenylpropanoid biosynthesis pathway (Supplementary Table S3). Ten DEGs involved in the phenylpropanoid biosynthesis and metabolism pathway were annotated as phenylalanine ammonia-lyase genes and were upregulated upon STS treatment. Among the plant hormone signal transduction pathways, 14 DEGs were assigned to auxin signaling, including five auxin-responsive genes, five auxin-induced genes, four auxin-transporter genes, and one auxin influx carrier-like gene; five DEGs were annotated as indole-3-acetic acid-amido synthetase GH3.1; and two DEGs were annotated as ethylene-responsive transcription factors, according to the annotations in the Swiss-Prot and Nr databases (Supplementary Table S3). In addition, some hormone-related DEGs were not included in the plant hormone signal transduction pathway, such as seven gibberellin synthesis and signaling DEGs, two abscisic acid-related DEGs, one jasmonate-related DEG, and 22 ethylene-responsive transcription factor genes. In the KEGG analysis, 17 of the top 20 pathways contained both upregulated and downregulated DEGs. The expression levels of 13 DEGs, involved in plant–pathogen interaction pathways, were upregulated after STS treatment. Four DEGs (CmoCh07G012020, CmoCh08G005780, CmoCh11G013620, and CmoCh12G000350) involved in stilbenoid, diarylheptanoid, and gingerol biosynthesis were also included in the flavonoid biosynthesis pathway, and expression levels of all were downregulated after STS treatment.

### Validation of RNA-Seq results using qRT-PCR

**FIGURE 5:**
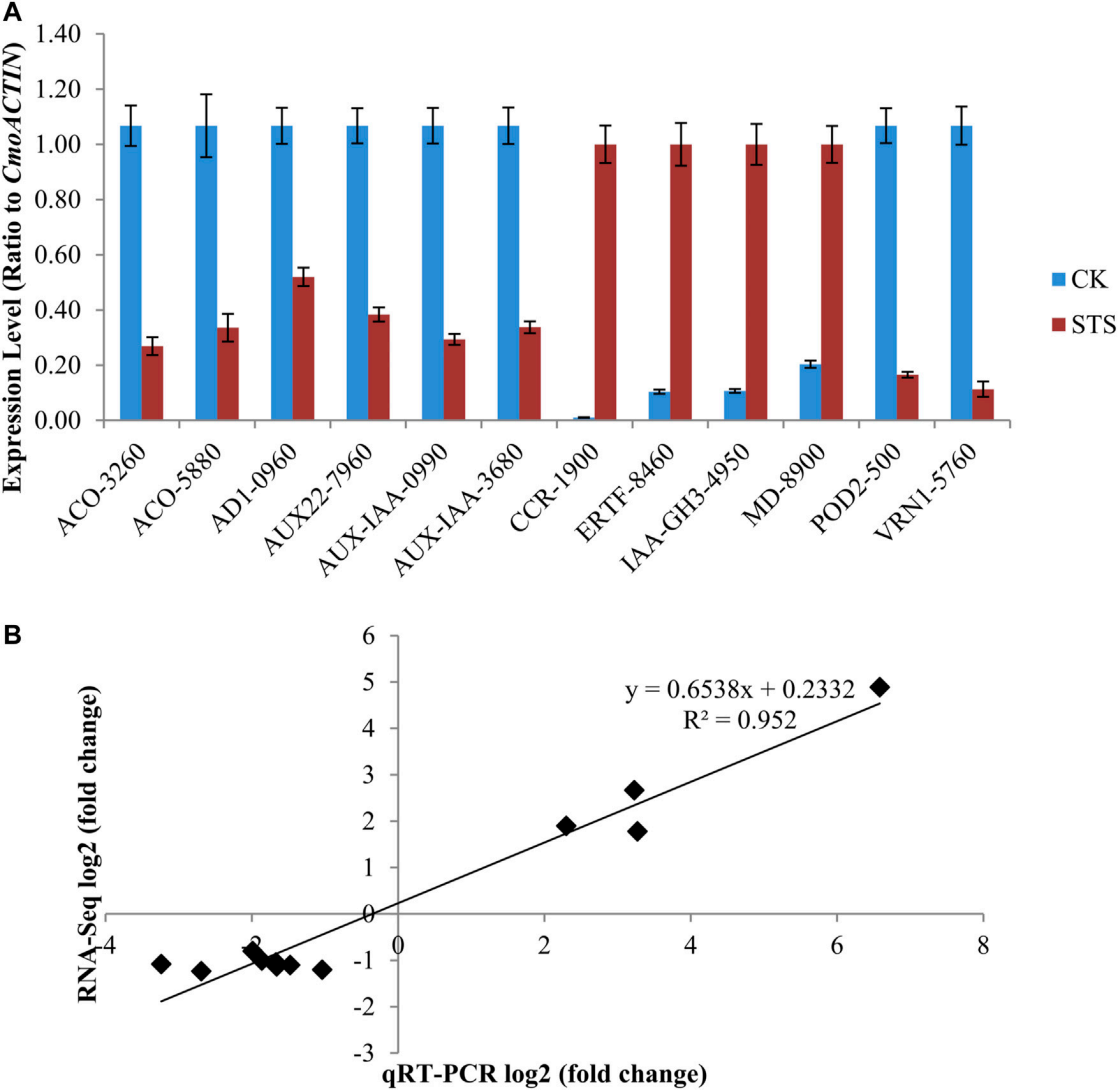
Comparison of the relative expression level change in 12 randomly selected differentially expressed genes (DEGs) by qRT-PCR and RNA-Seq. **(A)** Relative expression levels of genes in control and silver thiosulphate-treated plants. Values are the mean ± SE (standard error). **(B)** Correlation analysis of qRT-PCR log_2_ (fold-change) and RNA-Seq log_2_ (fold-change) values of the selected DEGs. ACO-5880 (CmoCh03G005880), AD1-0960 (CmoCh09G010960), AUX22-7960 (CmoCh17G007960), AUX-IAA-0990 (CmoCh07G010990), AUX-IAA-3680 (CmoCh03G003680), CCR-1900 (CmoCh01G001900), ERTF-8460 (CmoCh14G018460), IAA-GH3-4950 (CmoCh04G004950), MD-8900 (CmoCh03G008900), POD2-500 (CmoCh20G000500), and VRN1-5760 (CmoCh02G005760).

To determine the reliability of the Illumina HiSeq 2000 read analysis, 12 candidate genes with higher differential expression were selected, and their expression profiles were compared. The expression levels of the 12 DEGs were in good agreement with the RNA-Seq data, with a correlation coefficient (*R* ^*2*^) of 0.952 (Figures 5A,B). This result indicates the accuracy of the RNA-Seq data.

### Endogenous hormone response to STS treatment

The levels of endogenous hormones, including auxin, cytokinins (CKs), abscisic acid (ABA), jasmonates (JAs), salicylic acid (SA), gibberellins (GAs), ethylene (ETH), and strigolactones (SLs), in the shoot apical meristems of control and STS-treated plants were determined to evaluate the effects of STS. Hormone levels with a fold change ≥2 were considered different (Table 1). The levels of 2-oxindole-3-acetic acid (OxIAA, auxin), para-topolin riboside (pTR, CK), jasmonoyl-l-isoleucine (JA-ILE, JA), and dihydrozeatin-O-glucoside riboside (DHZROG, CK) were significantly decreased after STS treatment. The levels of cis-zeatin riboside (cZR, CK), trans-zeatin-O-glucoside (tZOG, CK), and salicylic acid 2-O-β-glucoside (SAG, SA) were significantly increased. No significant differences in the levels of other endogenous hormones were observed between STS-treated and control plants.

**TABLE 1:** Endogenous hormone levels in the shoot apical meristems of control and silver thiosulphate-treated plants.

	OxIAA	pTR	DHZROG	cZR	tZOG	JA-ILE	SAG
CK	3.35 ± 0.58A	0.98 ± 0.13A	0.53 ± 0.08A	0.20 ± 0.01B	0.08 ± 0.01B	0.47 ± 0.07A	1090.00 ± 113.58B
STS	1.56 ± 0.26B	0.30 ± 0.06B	0.23 ± 0.03B	0.41 ± 0.01A	0.25 ± 0.01A	0.20 ± 0.06B	7426.67 ± 742.32A

Note: Data are the means (ng/g) of three replicates ±SD. Different letters indicate a significant difference at the 1% level, as determined by Duncan’s new multiple-range test.

OxIAA, 2-oxindole-3-acetic acid; pTR, para-topolin riboside; DHZROG, dihydrozeatin-O-glucoside riboside; cZR, cis-zeatin riboside; tZOG, trans-zeatin-O-glucoside; JA-ILE, jasmonoyl-L-isoleucine; SAG, salicylic acid 2-O-β-glucoside.

## Discussion

Floral sex differentiation is very important for both monoecious and dioecious plants. Sex modification with chemicals can be an option to induce staminate flowers in gynoecious lines to maintain their progenies (Koyama, 2008; Yamasaki and Manabe, 2011). Ethylene regulates plant growth and development, fruit ripening, sex differentiation, and interactions with other hormones (Iqbal et al., 2017; Tao et al., 2018). Besides its beneficial effects, ethylene sometimes causes undesirable effects, such as the ageing and wilting of flowers, in flowering ornamental plants (Olsen et al., 2015). Ethylene perception is key to the driving functions of ethylene. Ethylene perception is involved in regulating floral sex differentiation and other actions related to ethylene (Duan et al., 2008; Wang et al., 2010). Thus, blocking ethylene responses is an efficient method to restrain the undesirable effects of ethylene and to induce differentiation into male flowers.

STS and AgNO_3_ are effective ethylene perception inhibitors. Previous studies have indicated that STS can be used to induce staminate flowers in monoecious and dioecious plants (Mishra et al., 2015; Lubell and Brand, 2018; Parimala et al., 2019), and increase flower production and longevity in flowering ornamental plants (Serek et al., 2015). Our results indicate that STS treatment delays the occurrence of the first female flower and increases the number of male flowers significantly. These results confirm the effects of STS on floral sex differentiation in monoecious plants, corroborating the results of previous studies (Mishra et al., 2015; Lubell and Brand, 2018).

Transcriptome analysis revealed that many DEGs between STS-treated and control plants are auxin signaling genes, which are involved in hormone signal transduction pathways, based on KEGG analysis. The application of silver ions can decrease indole-3-acetic acid (IAA) responsiveness and reduce Aux/IAA protein levels, promoting IAA efflux, and this effect is independent of the effects of silver ions on ethylene perception (Strader et al., 2009). Our results corroborate the results of this previous study, with the application of STS reducing the expression levels of auxin-transporter like protein genes and one auxin influx carrier-like protein gene and significantly decreasing the level of OxIAA. Many ethylene-responsive transcription factors were identified as DEGs; the expression of some of them was downregulated, whereas that of others was upregulated, under STS treatment. The reason for these results might be the fact that different ethylene-responsive transcription factor genes play different roles in the response to STS. As reported, the ERF family genes are involved in the ethylene response in a stress response-dependent or independent manner (Ohme-Takagi and Shinshi, 1995). Except for hormone signal transduction pathways, phenylpropanoid biosynthesis and metabolism pathways were the other predominantly enriched pathways related to the DEGs. Phenylalanine ammonia-lyase (PAL) catalyzes the first rate-limiting step in phenylpropanoid metabolism and provides precursors for a wide range of phenolic compounds, flavonoids, and lignins (Dong et al., 2016). Previous studies have found that PAL has very high specific activity in the anther tapetum, and a reduction in pollen fertility was observed in antisense and sense *PAL* transgenic tobacco (Rittscher and Wiermann, 1983; Matsuda et al., 1996). In this study, expression levels of 10 *PAL* genes were upregulated with STS treatment. These results indicate that *PAL* might be involved in male flower development. In addition, levels of 13 DEGs involved in plant–pathogen interaction pathways were upregulated, and those of SAG were increased after STS treatment. This could be related to the resistance response of pumpkin plants to silver ion stress during the process of inducing male flowers. Some DEGs, comprising *PALs* and *ERFs* might play a dual role in both inducing maleness and responses to silver ion stress. This requires further studies of gene function for confirmation. Thus, the regulatory mechanism of STS in the induction of maleness was found to be very complex. The effects of STS, including the transcriptional level influences of ethylene perception and IAA signaling genes, a series of hormonal interactions (Iqbal et al., 2017), and the responses to heavy metals (silver ions), might all play indispensable roles in the process of inducing the maleness of pumpkin.

## Conclusion

This study found that the application of STS to pumpkin at the seedling stage could significantly increase the number of male flowers, reduce the number of female flowers, and delay the occurrence of the first female flower. From the perspective of the transcriptome and endogenous hormone levels, STS treatment affects not only the transcription of auxin synthesis and signaling genes but also that of ethylene response factor genes and modulates the levels of auxin, JA, SA, and CKs, which together might contribute to the maleness of pumpkin. The results of the current study give insights into understanding the regulatory mechanism of STS with respect to the induction of maleness in pumpkin and they could provide production guidance for the induction of maleness using STS for the reproduction of gynoecious lines of Cucurbitaceae crops.

## Data Availability

The raw data are available in the National Center for Biotechnology Information (NCBI) BioProject database (accession number PRJNA838377).

## Abbreviations

DEGs, differentially expressed genes; qRT-PCR, quantitative real-time PCR; Nr, the NCBI non-redundant protein sequences; Nt, the NCBI non-redundant nucleotide sequences; Pfam, protein family; KOG, clusters of orthologous groups of proteins; KEGG, Kyoto encyclopedia of genes and genomes; GO, gene ontology; STS, silver thiosulfate; PAL, phenylalanine ammonia-lyase.
